# Designing AI tools to advance health equity in resource-constrained low- and middle-income countries

**DOI:** 10.1177/20552076261438662

**Published:** 2026-05-16

**Authors:** Anindya Pradipta Susanto, David Lyell, Bambang Widyantoro, Shlomo Berkovsky, Farah Magrabi

**Affiliations:** 1 Centre for Health Informatics, Australian Institute of Health Innovation, 7788 Macquarie University, Sydney, NSW, Australia; 2 Artificial Intelligence and Digital Health Research Group, Medical Technology Cluster, Indonesian Medical Education and Research Institute (IMERI), Faculty of Medicine, 64733 Universitas Indonesia, Jakarta, Indonesia; 3 Department of Medical Physiology and Biophysics, 64733 Faculty of Medicine Universitas Indonesia, Jakarta, Indonesia; 4 Department of Cardiology and Vascular Medicine, Faculty of Medicine Universitas Indonesia, 64733 National Cardiovascular Centre Harapan Kita Hospital, Jakarta, Indonesia

**Keywords:** artificial intelligence, global health, health equity, digital health, clinical decision support, resource-limited settings

## Abstract

In the resource-constrained settings, shortages of health workers and underdeveloped infrastructure hinder the delivery of equitable, high quality care. This perspective outlines strategic principles to support the design of AI tools that are genuinely beneficial in low-resource contexts: adopting problem-driven approaches, understanding the socio-technical context, selecting appropriate clinical tasks, and ensuring point-of-care accessibility, clinical comprehensibility, and actionable recommendations, ultimately improving clinicians’ decision-making and promoting health equity in low- and middle-income countries.

## Introduction

Artificial Intelligence (AI) is increasingly being embedded into clinical decision support (CDS) tools to improve care delivery and patient outcomes.^1–3^ Yet, adoption remains limited in environments where resources for high-quality healthcare are constrained.^1–3^ Key barriers include restricted access to specialist doctors and underdeveloped infrastructure.^4–7^ In such contexts, AI can be potentially transformative, expanding access to health services and strengthening systems that underpin health equity.^8,9^ By supporting better decision-making and optimising resource allocation, AI has the potential to improve access and reduce disparities across both developing and developed nations. Even in high-income countries, resource-constrained settings persist especially in rural and regional areas,^10^ Indigenous health services^11^ and safety-net organisations.^12^

While global health informatics has advanced in developed nations and tertiary centres, there remains limited literature on the design of clinician-facing AI tools in resource-constrained settings.^13^ A recent review found that 91% of AI evaluation studies in clinical settings were conducted in secondary or tertiary care environments, and all were undertaken in high-income countries.^1^ This gap limits the contribution of AI to equitable health outcomes globally. Recent contributions have focused on human-AI interaction,^14^ frameworks for AI-based clinical trials,^15^ and guidance for translating AI into tertiary academic centres.^16^ This perspective aims to outline approaches to designing clinician-facing AI tools through a socio-technical lens, with the aim of advancing health equity in resource constrained low- and middle-income countries (LMICs). We outline eight principles to support safer and more effective AI tools in these contexts. These principles extend beyond the foundational requirements of high-quality datasets, model performance, and model transportability, which continue to challenge tool development across the AI lifecycle.^8^

## Eight principles for designing AI tools for resource-constrained clinical settings

### Take a problem-driven approach

AI system design frequently prioritises the availability of annotated datasets and model performance metrics—priorities that are often misaligned with the operational realities of resource-constrained settings. The approach to AI use in resource-constrained settings should be problem-driven, guided by care delivery needs. These settings typically face three interrelated challenges: inequitable access to care due to workforce shortages, inadequate health infrastructure, and the dual burden of infectious and chronic diseases. Together, these underscore the urgent need for stronger preventive approaches. This contrasts with high-income nations, where AI adoption has historically been top-down and technology-driven, supported by well-coordinated health systems, state-of-the-art information infrastructure, and interoperability standards.^17^

The challenges in LMICs are complex and interconnected, requiring multi-pronged solutions. Yet, AI may often be perceived as a silver bullet. A problem-driven approach helps refocus attention on specific clinical needs, treating AI as one of many possible interventions and enabling comparison with alternative approaches. When AI is identified as a viable option, model design and selection should be guided not by technology-driven performance but by alignment with the socio-technical context and the task at hand.

The problem-driven approach to public health challenges is evident in recent studies, particularly in contexts where decision-making is suboptimal. For instance, AI screening tools for infectious diseases (e.g. tuberculosis and HIV) in Malawi and chronic diseases (e.g. diabetic retinopathy) in Bangladesh.^18,19^ Both AI tools were evaluated in primary care to improve the productivity of preventive care by 40%, address limited access to specialist doctors,^18^ enhance the timeliness of interventions, and increase cost efficiency.^19^

### Examine the socio-technical context for introducing AI

**Figure 1.**
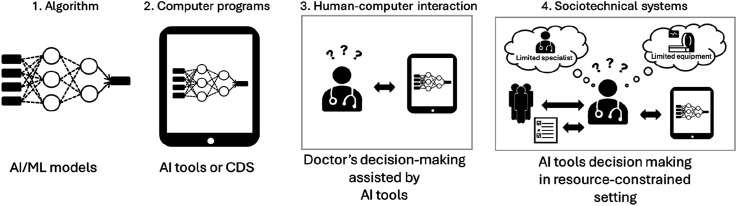
Four layers of informatics systems nest within each other (algorithm> computer programs> human-computer interaction> sociotechnical systems), adapted from Coiera (2015). AI/ML algorithms are embedded in software tools. Doctors interact with AI tools within resource-constrained settings where access to specialist and medical equipment is limited. Abbreviations: AI, artificial intelligence; CDS, clinical decision support; ML, machine learning.

Improving the design and implementation of new technologies, including AI, requires integrating socio-technical considerations to ensure social and technical elements are effectively aligned. As defined, “the socio-technical view attempts to understand the contribution of phenomena at the human social level to the performance of technical systems.”^20^ In healthcare, this means AI must be designed and evaluated within the complex socio-technical context in which it operates, particularly in relation to the clinicians who use it. As illustrated in Figure 1, informatics interventions are embedded across multiple, interacting system layers,^21^ with clinicians occupying a central role within the socio-technical system.

“The technical systems have social consequences, and social systems have technical consequences.”^22^ While AI offers significant potential to enhance healthcare quality and safety, its integration into clinical workflows can introduce risks that may compromise patient safety.^23^ The inseparability of social and technical elements makes socio-technical analysis essential in AI design and implementation. This includes careful consideration of the socio-technical context, particularly in settings with constrained resources and competing demands.^20^ By explicitly incorporating socio-technical considerations, AI can evolve from a purely automated tool into a socio-technical system that mitigates patient safety risks and supports responses to emerging threats.

One way to understand the socio-technical context of AI tools and their impact within a specific health system is through systems engineering. Frameworks such as SEIPS (Systems Engineering Initiative for Patient Safety) provide structured methods for examining this context.^24^ SEIPS has been widely applied in health-system research and is closely tied to patient safety, a core dimension of modern health systems. For example, a qualitative study to examine clinicians use of AI tools can apply SEIPS to describe the work system: people, environment, tasks, and technology; within specific processes and outcomes, such as AI-based cardiovascular disease prediction in resource-constrained clinical settings.^25^

### Automate the right clinical task

While AI aims to extend clinician capabilities beyond what is possible unaided,^26^ a critical question remains: which tasks should be automated?^27^ AI must target the right tasks for a given clinical problem and socio-technical context. Assessing the work system, processes, and intended outcomes determines a task’s suitability for AI automation. For instance, in LMICs, available inputs may be limited to clinical data and basic laboratory tests. A user-centred approach is essential to validate appropriate clinical tasks,^28^ ensuring alignment with user needs and minimizing workflow disruption. At the same time, a meaningful workflow adjustment is usually required for AI tools to function reliably, for instance, introducing a more structured or systematic data entry. As such, there is a mutual adjustment where both AI and the traditional workflow evolve to achieve safe and effective socio-technical integration.

In clinical practice, the most common clinical tasks of AI include imaging interpretation and predictive risk assessment, particularly for early sepsis detection.^1,29^ Among these, AI has been reported to achieve its greatest success in clinical documentation and risk assessment.^29^ In their real-world evaluation, a limited number of AI tools have shown potential to automate imaging interpretation for screening in remote or underserved areas, including applications in hepatobiliary disease,^30^ childhood cataracts,^31^ and skin lesion.^32^

### Ensure AI tools are easily accessible at the point of care

AI tools should be delivered via mobile devices as they are widely available in resource-constrained settings and operable by users with basic digital literacy.^25,33^ In LMICs, clinical decision support tools are most commonly accessed via personal mobile devices.^25^ Smartphones are widely used for clinician communication and consultation, with data entry frequently performed by patients or non-physician health workers prior to physician review.^25^ A recent review reported that communication (90%) and clinical decision-making (70%) were the predominant uses of smartphones by clinicians, including in LMIC contexts.^34^ Increasing mobile processing power now enables on-device AI, and mobile-based tools can be designed to function offline, reducing reliance on internet connectivity.

### Ensure use of AI can improve decisions

Given there is limited access to confirmatory examinations, clinical decision-making is largely subjective in resource-constrained settings, with clinicians relying on their own experience or peer consultation.^25^ The implementation of AI tools in these contexts should evaluate their impact on improving clinical decision-making. As such an AI-assisted clinician must perform better than the same clinician unaided. Given that most contemporary AI tools are assistive, leaving the final decision to the clinician, the interaction between clinician and AI is fundamental to improved decision-making.^1,3,35^ Consequently, comparing AI model performance against clinicians alone is an inadequate metric, as it fails to capture outcomes when both work together. Evaluating clinical decision-making is a necessary precursor to demonstrating positive impacts on care delivery and patient outcomes.^36^

Evidence of enhanced clinical decision-making can first be evaluated in controlled settings and subsequently in real-world environments after deployment. To evaluate improvements in decision-making, randomised controlled trials (RCTs) should be used, comparing the accuracy of AI-assisted versus unassisted clinicians.^27^ However, few RCTs focus on decision-making itself, with most measuring downstream effects on care delivery and patient outcomes.^2^ For instance, a real-world RCT assessing AI support during emergency calls demonstrated no significant difference in operators’ ability to recognise cardiac arrest.^37^ On the other hand, clinical vignette studies offer a way to evaluate decision-making in controlled settings and to show the safety and efficacy of AI-assistance prior to costly and potentially disruptive trials.^38^ As risk- and patient-free designs, they mitigate potential harm from early AI experimentation.^39^

### Consider time costs: Expediting diagnosis and treatment

In resource-constrained LMICs, clinicians face high patient volumes and limited time per case, making time arguably the scarcest resource.^25^ This challenge is compounded by manual, repetitive tasks arising from the lack of electronic medical records. Contemporary assistive AI tools require clinicians to review, verify, and approve or reject AI-generated recommendations.^1,3^ To be beneficial, AI must not add to clinician workload or delay diagnosis and treatment. Instead, AI-assisted decision support should provide timely recommendations that expedite rather than hinder time-sensitive decisions.^40^

Accordingly the time costs associated with the use of AI tools for clinical decision-making should be evaluated.^41^ For example, AI-assisted ECG triage that operate in parallel with cardiologist confirmation, could substantially reduce door-to-balloon time for patients with myocardial infarction.^42^ A previous RCT of childhood cataract screening reported faster diagnosis with AI support compared to clinicians alone (2.79 min vs 8.52 min, *p*<0.001).^31^ Time-sensitive AI has the potential to alleviate, rather than worsen, delays in diagnosis and treatment common in resource-constrained settings. As noted in the “ten commandments” for CDS, “speed is everything.”^43^ Conversely, AI that slows clinical processes risks becoming a form of clumsy automation, leading to disuse.^44^

### Ensure AI recommendations are clinically comprehensible through explanation

AI tools should provide explanations to help clinicians contextualise their output and support decision-making. Clinicians typically integrate multiple sources of information through clinical reasoning while attempting to interpret the “black-box” outputs of AI.^45^ This preserves their decisional discretion when considering AI recommendations. In high-resource settings, clinicians can contextualise AI outputs with additional diagnostic tests or subspecialist opinions. By contrast, in LMICs, objective data for confirmatory diagnosis are often limited. This makes explainability critical, AI outputs must be interpretable to support safe clinical decision-making.^46^ Studies have shown higher clinician acceptance and more positive perceptions when AI results are clinically comprehensible.^47^ For example, studies of AI tools for pulmonary embolism risk assessment have shown that model-agnostic local explanations highlighting individual risk factors help clinicians interpret and trust AI outputs, while also enabling them to explain results to patients.^48^ AI should support clinicians in providing meaningful explanations, thereby facilitating shared decision-making.^49^

### Ensure AI recommendations are actionable to impact care delivery

This principle highlights the importance of designing AI solutions that both meet genuine needs and generate outputs that are actionable within the contextual constraints of their intended settings. For AI tools to be valuable, clinician decisions informed by AI must translate into concrete actions.^50^ Changes in patient management can then serve as indicators of actionable care delivery.^1^ For example, following AI-based risk assessment for atherosclerotic cardiovascular disease, actionable care may involve prescribing primary preventive therapies such as aspirin or statins. The actionability of AI-assisted decision-making can be further evaluated through net benefit analysis.^51,52^

Conversely, predictive AI-based risk assessments that lack actionable outputs provide little value. For instance, an AI tool distinguishing ischemic from haemorrhagic stroke showed strong performance in prioritising air ambulance transport, thereby avoiding treatment delays and unnecessary deployments.^53^ Yet, in regions without emergency helicopter services, such a tool would have minimal impact on care delivery or outcomes. AI implementation in resource-constrained LMICs must be responsible and sustainable, ensuring it does not exacerbate existing burdens or introduce new ethical challenges.^54^ Treatment options in these contexts are often limited by shortages of essential medicines or specialised equipment. Accordingly, AI output must be tailored to what is realistically achievable in the specific setting. At the deployment stage, AI tools should be governed by clear clinical consensus and locally grounded policies, supported by ethics evaluation to ensure transparency and accountability.^55^ Such governance enhances actionability while reducing the risk of unintended or adverse effects on care delivery.

## Case study: Cardiovascular disease risk assessment

Using the eight socio-technical principles provides a pragmatic, structured approach to design AI tools for resource-constrained settings. To synthesise these principles, we present an example case study in risk assessment for Atherosclerotic Cardiovascular Disease (ASCVD) which is a priority in global health (Table 1). The case study focuses on resource-constrained settings in Indonesia as the socio-technical context where primary care doctors play an important role in preventing ASCVD by assessing and managing cardiovascular risk factors. It was drawn from previous works including a literature review,^1^ and semi-structured interviews which examined the socio-technical context of cardiovascular risk assessment with limited access to medical specialists and advanced equipment (principles 1-4).^25^ These requirements were then used to construct a conceptual prototype of an AI tool that was demonstrated to improve risk assessment and statin prescriptions, and reduced decision-making time (principles 5-8).^56^ We show that ASCVD risk assessment is a promising application for AI in resource constrained settings, and merits further evaluation to assess real-world impact on clinical decision-making and patient management.

**Table 1.** Applying the eight principles to design and test an AI tool for ASCVD risk assessment in Indonesia.

Principles for designing AI for resource-constrained clinical settings		Case study in ASCVD risk assessment^1,25,56^
1. Problem-driven	Take a problem-driven approach	Problem: ASCVD is the most common cardiovascular problem placing significant clinical, social, and financial burdens on the Indonesian health system. Potential users i.e. doctors working in resource-constrained settings were interviewed to discuss and confirm ASCVD risk prediction as a compelling use case for an AI tool to improve primary prevention.^25^
2. Socio-technical context	Examine the socio-technical context for introducing AI	The clinical work system in resource-constrained settings was examined using SEIPS, a theoretical framework to describe complex sociotechnical systems including people, processes, tasks, and technology.^24^ Doctors faced substantial patient volumes. Therefore, time was a significant constraint. They relied predominantly on paper-based medical records, often made clinical decisions based on subjective judgement due to limited access to confirmatory diagnostic investigations, such as specialised laboratory tests or imaging.^25^
3. Right task	Automate the right clinical task	Risk assessment represents a key application for AI in healthcare (28%).^1^ Doctors reported needing support to prioritise high-risk patients and deliver patient education tailored to risk level.^25^
4. Easy access	Ensure AI tools are easily accessible at the point of care	A prototype AI tool was developed and tested.^56^ It was designed to be accessible on a mobile device which is a likely real-world implementation given the widespread availability and use of mobile phones among doctors, including for communication and consultation with colleagues regarding clinical cases.^25^
5. Improve decisions	Ensure use of AI can improve decisions	A randomised controlled clinical vignette study was undertaken with 102 doctors to compare the prototype tool with the standard care for risk assessment without decision support, please see detailed methods in the reference.^56^ Use of the AI tool was shown to improve ASCVD risk assessment by 27% (*p*<.001), equivalent to one additional correct risk classification for every four patients assessed with AI support.
6. Time costs	Consider time costs: expediting diagnosis and treatment	Use of the prototype AI tool reduced the time required for ASCVD risk assessment by doctors by 12% compared with standard care.^56^ Decision-making assisted by AI remained faster than heuristic risk assessment without decision support.
7. Explanation	Ensure AI recommendations are clinically comprehensible through explanation	Doctors emphasised the need for AI tools to provide explanations of input factors and risk scores, rather than only risk level recommendations.^56^
8. Actionable	Ensure AI recommendations are actionable to impact care delivery	AI recommendations were considered actionable given the availability of different options for prescribing statins. Prescription of statins (*p*<.001) and referrals was also improved (*p*=.029).^56^

*Note.* AI, artificial intelligence; ASCVD, atherosclerotic cardiovascular disease.

The case study was confined to ASCVD risk prediction in Indonesia. Further research applying diverse AI tools across LMICs with varying clinical governance structures is needed to assess the generalisability of these principles. Alternatively, these principles could inform a desktop simulation to evaluate whether a proposed AI tool would yield material benefit in resource-constrained settings, with Principles 1 and 8 serving as the primary evaluative criteria.

## Conclusion

Resource-constrained settings face profound challenges in delivering equitable, high-quality healthcare. AI has the potential to alleviate some of these pressures, but only if designed with contextual sensitivity. This perspective highlights key principles for responsible AI in LMICs: grounding development in problem-driven approaches, embedding socio-technical understanding, prioritising appropriate clinical tasks, and ensuring tools are accessible, comprehensible, and actionable at the point of care. By aligning AI design with local realities, clinicians’ decision-making can be strengthened and health equity meaningfully advanced across LMICs.

## Data Availability

No datasets were generated or analysed during the current study.*

## Appendix

### Abbreviations

AI: Artificial intelligence
ASCVD: Atherosclerotic cardiovascular disease
CDS: Clinical decision support
ML: Machine learning.
